# The History and Capabilities of Herbal Simples

**Published:** 1890-11-22

**Authors:** W. T. Fernie


### The History and Capabilities of Herbal Simples.

By W. T. Fernie, M.D.

### XXIII.—COWSLIP.

Quite early in the year our English pastures and meadows—especially where there is a clay soil of blue lias—become coyly clothed with pale primroses, and more brilliantly gay "with gaudy cowslips drest." But it is quite a mistake to suppose that the latter flowers are favourite food with cows, who, in fact, never eat them if they can help it. The name, says Dr. Prior, is really derived from two Flemish words—kous loppe—meaning hose-flap, a very humble part of woollen nether garments. Originally both the Mullein, or Adam's flannel, which has large oval woolly leaves, and the Cowslip were included under one common Latin name, Verbascum, by reason of which the attributes of the Mullein still remain accredited in mistake to the cowslip. Yorkshire people call it the cowstripling; and in Devonshire, where the flower is scarcely to be found because of the red marl, it has come about that the foxglove goes by the name of cowslip. In many provincial districts the cowslip is known as the Petty Mullein; and in others as Paigle, or Palsy wort. The old English proverb, "as blake as a paigle," means as yellow as a cowslip. Or, it may be that the term has to do with the Dutch word spiegel, a mirror, in reference to children amusing themselves by reflecting the flower upon the chins of their playmates to see if they love butter. But former medical writers called the cowslip "herba paralysis," or palsy wort, because of its supposed efficacy in relieving paralysis; and the whole plant is known to be gently narcotic, and somniferous. Physicians for the last two centuries have used the powdered roots of the cowslip and of the primrose for wakefulness, hysterical attacks, and muscular rheumatism; and the cowslip root bore of old the names *radix arthritica* and *radix paralyseos*. Both the root and the flowers have an odour of anise, which is due to a small quantity of a volatile oil identical with mannit. Their more acrid principle is saponin. Pliny says, "In aqua potum omnibus morbis mederi tradunt." And Hill tells us, that when boiled in ale the roots are taken by country persons for giddiness of the head with frequent and happy success. "They be likewise in great request amongst those that use to hunt after goats and roebucks upon high mountains, for the strengthening of the head when they pass by fearful precipices and steep places in following their game, that giddiness and swimming of the brain may not seize upon them." The dose of the dried and powdered flowers is from fifteen to twenty grains. A syrup of a fine yellow colour may also be made from the flowers which answers a like purpose. Three pounds of the fresh flowers should be infused in five pints of boiling water, and then simmered down to a syrup with sugar. Pope praised the herb and its flowers on account of their sedative qualities;

"For want of rest
Lettuce and cowslip wine - probatum est."

This fragrant and pleasant brew, concocted from the blossoms with lemons and sugar, has long been one of the most esteemed articles of the domestic pharmacopœia. Coleridge makes Christabel declare

"It is a wine of virtuous powers;
My mother made it of wild flowers."

Botanically the cowslip has arrogated to itself the name of "primula veris," the first flower of spring, which more properly belongs to the primrose. In the language of flowers it signifies youthful beauty. The tiny people were supposed in Elizabethan times to love to nestle in the drooping bells of cowslips, and hence the flowers were then called fairycups. The "five small drops of red in the golden chalice shed," were said to possess the rare virtue of retaining for youth its beauty, or of restoring it when lost; also of removing freckles from the face, according to the doctrine of signatures:

"In their gold coats spots you see;
These be rubies; fairy favours,
In these freckles live their savours."

Herbals of the same date say that an ointment made of cowslip flowers "taketh away the spots and wrinkles of the skin, and doth add beauty exceedingly, as divers ladies, gentlewomen, and she citizens—whether wives or widdows—know well enough!" The flowers of the cowslip may be made into a delicate conserve; and cowslip tea infused from the leaves is very quieting against nervous disturbances. These leaves were at one time eaten in salad, and mixed with other herbs to stuff meat. The cluster of blossoms on a single stalk sometimes bears the name of lady's key, or of St. Peter's wort, either because it resembles a bunch of keys, or because it unlocks the treasures of spring, and first reveals its stores of gold and jewels. In Somersetshire cowslip flowers are done up by playful children into balls, which they call tisty tosty, or sometimes simply a tosty. For this purpose the umbels of blossoms fully blown are strung closely together and tied into a firm ball. In conclusion a word should be said in favour of the poor cow with whom I have dealt so unsentimentally as concerns the cowslip. The breath and smell of this sweet-odoured animal are thought in Fifeshire to be good against consumption. And Henderson tells of a blacksmith's younger apprentice who was at length restored to health when far advanced in a decline by eating butter made from the milk of cows fed in a kirkyard. In the south of Hampshire a plaster of warm cow-dung is usefully applied to open wounds. Furthermore, in its evolutionary development of detail the large homely animal reads us a lesson. "Dat Deus immiti cornua curta bovi," says the Latin proverb, "savage cattle have only short horns." So was it in "the house that Jack built," where the fretful creature that tossed the dog had but one horn, and that, we are told, grew crumpled.

